# Quality by Design Approach to the Optimization of Cohesive Powder Blending in Direct Compression

**DOI:** 10.3390/pharmaceutics18070823

**Published:** 2026-07-02

**Authors:** Mateusz Przywara, Patryk Leszczak

**Affiliations:** 1 Department of Chemical and Process Engineering, Faculty of Chemistry, Rzeszow University of Technology, Powstańców Warszawy 6, 35-029 Rzeszów, Poland; 2 Doctoral School, Rzeszow University of Technology, Akademicka 2, 35-084 Rzeszów, Poland

**Keywords:** quality by design, direct compression, process optimization, response surface methodology, cohesive powders

## Abstract

**Background/Objectives:** Direct compression of cohesive powders is often challenged by poor flow, blend heterogeneity, and variable tablet quality. This study investigated how mixing time, fill level, and rotational speed affect the blending behavior and tablet properties of a sodium naproxen–calcium carbonate formulation and aimed to define a robust operating space for process optimization. **Methods:** Powder blends were prepared in a V-type mixer according to a central composite design and analyzed using response surface methodology. The effects of the three process parameters were evaluated through powder flow descriptors (angle of repose, angle of fall, and angle of difference) and tablet quality attributes, including thickness, mass, active pharmaceutical ingredient (API) content, and abrasiveness. Statistical significance was assessed by ANOVA, and a design space was established using predefined acceptance criteria. **Results:** Mixing time significantly affected the angle of difference, indicating changes in blend cohesiveness and flow uniformity, whereas fill level significantly influenced API content. Tablet thickness and mass remained relatively stable across the tested conditions. Abrasiveness showed the greatest numerical variability and tended to increase at high fill levels combined with short mixing times. Response surface analysis identified two acceptable operational regions that satisfied the quality criteria for blend homogeneity, API content, and abrasiveness. **Conclusions:** The studied process variables exerted selective, property-specific effects rather than uniform changes across all quality attributes. The results support QbD-based process design for cohesive direct-compression systems and show that robust tablet manufacture can be achieved within more than one operating window.

## 1. Introduction

Direct compression (DC) is an attractive manufacturing method for solid oral dosage forms due to its simplified process flow, elimination of granulation steps, and potential for improved production efficiency. From a chemical engineering perspective, however, DC is highly sensitive to powder material properties, as unit operations such as feeding, conveying, blending, and die filling are performed without prior modification of particle structure. Consequently, powder flowability, cohesiveness, and compressibility directly govern process stability as well as critical quality attributes of the final tablets, including mass uniformity, content uniformity, and mechanical strength [1,2,3].

Sodium naproxen is a representative active pharmaceutical ingredient exhibiting particularly unfavourable processing characteristics, including fine particle size, high specific surface area, limited flowability, and poor compactability [4]. Similar challenges are associated with fine-grade calcium carbonate, widely used as a mineral filler, whose bulk behaviour is dominated by strong interparticle interactions rather than frictional forces [5]. Rheological studies of pharmaceutical powders consistently demonstrate that cohesion is the primary factor controlling bulk flow and handling behaviour, outweighing the influence of particle shape or friction coefficients [6,7]. As a result, blends of sodium naproxen with calcium carbonate represent one of the most demanding material systems for direct compression, especially at high drug loadings, where stable die filling and reproducible tablet mass become increasingly difficult to achieve [1,3].

From a powder engineering standpoint, mixing technology and the amount of mechanical energy introduced into cohesive systems are critical determinants of blend performance. Early fundamental studies on cohesive powder mixing highlighted the narrow operational window between insufficient dispersion and excessive energy input leading to segregation or particle structure alteration [8]. More recent investigations have shown that appropriately selected high-intensity mixing techniques, including vibratory mixing, can significantly improve content uniformity and compact mechanical strength in direct compression, even for extremely poorly flowing materials [9]. Such effects have also been reported for sodium naproxen systems combined with carbonate-based mineral fillers, where short, well-controlled mixing times enabled tablet quality comparable to that obtained via wet granulation, despite unfavourable raw-material flow properties [10].

To mitigate the adverse effects of powder cohesion on individual unit operations, a range of material and process engineering strategies has been proposed. These include the selection of excipients with favourable flow properties, the use of glidants, pre-blending approaches, and particle engineering techniques such as agglomeration, spray drying, or surface modification [3,11,12,13]. However, the effectiveness of these strategies is highly system-specific and strongly dependent on interactions between material attributes and process parameters, which complicates scale-up and the design of robust operating conditions [1,6]. In this context, Quality by Design (QbD) has increasingly been adopted as a structured framework for analysing and designing complex particulate processes. When interpreted from an engineering rather than a regulatory perspective, QbD provides a systematic approach to linking material properties, operating parameters, and product quality through risk analysis and experimental design [14,15,16]. The application of such tools enables identification of critical material attributes and process parameters governing mixing, flow, and compaction behaviour [17,18]. Related work in QbD-based blending and continuous direct compression also shows the value of design-space definition, predictive modelling, and process intensification, particularly when handling cohesive powders and variable raw materials [19,20,21].

In recent years, process analytical technologies and data-driven modelling have further expanded the QbD toolbox. Near-infrared spectroscopy and multivariate modelling have been used to monitor blend homogeneity, assess raw-material variability, and support adaptive process control in cohesive powder systems [1,22]. Likewise, continuous direct compression platforms and semi-continuous mini-blending approaches have demonstrated that robust performance at larger scale is possible, provided that process parameters are carefully tuned and material variability is accounted for [20,21,23]. These developments are particularly relevant for poorly flowing systems, where small changes in formulation or process conditions can strongly affect blend quality and downstream tablet performance.

Despite growing interest in this approach, its application to strongly cohesive powder systems combining poorly flowing active pharmaceutical ingredients with mineral fillers remains limited. Although numerous studies address sodium naproxen, calcium carbonate, powder mixing technologies, and QbD frameworks individually, there is a lack of integrated investigations that combine these aspects within a unified chemical engineering perspective on direct compression. In particular, the influence of mixing energy and mixing time on blend homogeneity, flowability, and compactibility of sodium naproxen–calcium carbonate systems, and the resulting implications for process stability and product quality, remain insufficiently understood. The present study aims to address this gap.

## 2. Materials and Methods

### 2.1. Materials

The formulation was composed of sodium naproxen (Divi’s Laboratories, Hyderabad, India), calcium carbonate (Chempur, Piekary Śląskie, Poland), microcrystalline cellulose (Avicel^®^ PH102, FMC BioPolymer, Philadelphia, PA, USA), polyvinylpyrrolidone (PVP), from Sigma-Aldrich, St. Louis, MO, USA and Aerosil^®^200 (Evonik, Essen, Germany). All raw materials were of analytical grade. The blend consisted of 20 wt.% sodium naproxen, 68 wt.% calcium carbonate as the main diluent, 8.5 wt.% microcrystalline cellulose serving as filler and disintegrant, 3 wt.% PVP as binder, and 0.5 wt.% silica, Aerosil^®^200 (Evonik, Germany) acting as a glidant (flow aid) and anti-caking agent to improve powder flow properties.

### 2.2. Methods for Determining the Properties of Raw Materials, Blends, and Tablets

The characteristics of raw powders and formulation blends were evaluated using a pharmacopoeia-compliant powder tester (PT-S Powder Tester, Hosokawa Micron B.V., Doetinchem, The Netherlands). The analyses comprised determination of the angle of repose, angle of fall, and angle of difference. The angle of repose was defined as the angle formed between the horizontal plane and the slope of a freely formed powder cone. The angle of fall was defined as the angle of the powder cone measured after the application of controlled vibrations, corresponding to three automatic impacts of the instrument hammer on the measuring platform, following the ASTM D6393-14 standard [24] for dry powder samples. The angle of difference was calculated as the difference between the angle of repose and the angle of fall. This parameter is commonly used as an indicator of powder flowability and cohesiveness, where higher values indicate a greater tendency of the powder bed to collapse under mechanical disturbance and are associated with increased powder mobility and flooding tendency. Particle size of the raw materials was assessed by laser diffraction with wet dispersion (Mastersizer 2000 Hydro MU, Malvern Instruments, Malvern, UK), performed in accordance with ISO 13320-1:1999 [25].

Tablet weight was measured using an analytical balance (Pioneer PX224, Ohaus, Greifensee, Switzerland), while thickness was determined with a digital caliper (0.01 mm resolution). Twenty tablets were randomly selected from each experimental batch. The weight and thickness of each tablet were measured in quadruplicate, and the mean value obtained for each tablet was used for further statistical analysis. All determinations were performed in quadruplicate. Tablet abrasiveness was examined using a rotating drum mixer (CE 245, GUNT, Barsbüttel, Germany). Approximately 50 g of tablets was subjected to 200 rpm rotation for 5 min in a 1.15 dm^3^ drum. The residues collected on a 500 µm sieve were weighed, and abrasiveness was expressed as the percentage of the initial mass. Each test was conducted in four replicates. Unlike the pharmacopeial friability test, the applied procedure was intended to provide intensified mechanical stress conditions, enabling discrimination between formulations exhibiting relatively small differences in mechanical resistance. The obtained mass loss was used as a comparative measure of tablet abrasiveness during process optimization.

The content of the API was quantified using a laboratory refractometer (RX 5000, ATAGO CO., Tokyo, Japan). Calibration samples were prepared using the complete excipient matrix corresponding to the tablet formulation and accurately weighed increasing amounts of sodium naproxen dispersed in 50 mL volumetric flasks filled with distilled water. The samples were shaken for 1 h to ensure complete dissolution of sodium naproxen and PVP. Insoluble components, including calcium carbonate, microcrystalline cellulose, and colloidal silicon dioxide, were subsequently removed by filtration through 0.2 µm syringe filters. The refractive index of each filtrate was measured at 20 °C. A calibration curve was established between sodium naproxen concentration and refractive index, yielding a coefficient of determination of R^2^ = 0.992.

For API determination, ten tablets were randomly selected from each experimental batch and individually weighed. Each tablet was transferred into a 50 mL volumetric flask containing 50 mL of distilled water and shaken at 120 rpm for 24 h to ensure complete dissolution of sodium naproxen and PVP. The resulting suspensions were filtered through 0.2 µm syringe filters and analyzed under the same conditions as the calibration samples. Sodium naproxen content was calculated from the calibration curve based on the measured refractive index values. Individual tablets were placed in 50 mL round-bottom flasks, subjected to continuous agitation for 24 h, and filtered through 2 µm syringe filters prior to measurement of the refractive index. Ten replicates were performed to ensure analytical reliability. Since the calibration standards contained the same excipient matrix as the analyzed samples, potential matrix effects on the refractive index measurements were inherently accounted for in the calibration model.

### 2.3. Preparation of Powder Blends and Tablets

Powder mixtures were homogenized in a V-type tumble mixer (chamber capacity 750 cm^3^, CDK, Gliwice, Poland). Tablet compaction was performed using a manual single-punch press TDP 0 (LFA Machines Oxford LTD, Oxfordshire, UK). Compression was carried out with 6 mm concave punches with beveled edges, the upper punch containing a score line to enable subdivision. Each tablet was produced under a constant compression force of 3.5 kN.

### 2.4. Quality by Design Framework

A QbD framework was applied to systematically evaluate the influence of process parameters on blend and tablet quality attributes. The approach was based on identification of critical process parameters (CPPs), including mixing time, fill level, and rotational speed, and their relationship with critical quality attributes (CQAs) of the final product. The investigated CQAs included powder flow characteristics expressed by the angle of repose, angle of fall, and angle of difference, as well as tablet thickness, mass, active pharmaceutical ingredient (API) content, and abrasiveness.

A face-centered central composite design (FCCCD) combined with Response Surface Methodology (RSM) was employed to establish quantitative relationships between process variables and measured responses. Experimental results were analyzed using second-order polynomial models to identify the main, interaction, and quadratic effects of the investigated parameters. Statistical significance of the factors was evaluated using analysis of variance (ANOVA).

Based on the obtained models, a design space defining acceptable operating conditions was established according to predefined quality criteria for blend homogeneity, API content uniformity, and tablet abrasiveness. The QbD framework enabled systematic assessment of process robustness and supported identification of operating regions ensuring reproducible product quality during direct compression processing.

### 2.5. Use of Generative Artificial Intelligence

Generative artificial intelligence (GenAI) tools were used as auxiliary tools during the preparation of this study. GitHub Copilot (GitHub, Inc. GitHub, Inc., San Francisco, CA, USA, version current at the time of use, https://github.com/features/copilot, accessed on 23 June 2026) was used to assist in the development and debugging of MATLAB (MathWorks, Natick, MA, USA, R2026a) scripts employed for response surface methodology (RSM) and design of experiments (DoE) analyses. ChatGPT (OpenAI, San Francisco, CA, USA, GPT-5, https://chatgpt.com) and Consensus (Consensus AI, Boston, MA, USA, version current at the time of use, https://consensus.app) were used to support literature exploration and the interpretation of results. All generated suggestions, code fragments, and outputs were critically reviewed, verified, and, where necessary, modified by the authors. The authors take full responsibility for the study design, data analysis, interpretation of results, and the final content of the manuscript.

## 3. Results

### 3.1. Raw Materials

The first step in the project was to investigate the properties of raw materials for tablet production. The properties of the active pharmaceutical ingredient (sodium naproxen) and the excipients used in the formulation are summarized in Table 1. Flow properties were assessed by measuring the angle of repose, angle of fall, and angle of difference, which provide insight into powder cohesiveness and flowability. Sodium naproxen and microcrystalline cellulose exhibited relatively high angles of repose (45.9° and 44.6°, respectively), indicating moderate cohesiveness and potential challenges in flow during handling. In contrast, calcium carbonate displayed a lower angle of difference (13.3°), suggesting better flowability and confirming its suitability as a primary diluent in tablet formulations. PVP showed a small particle size (d(0.5) = 21 µm) and moderate flow angles, consistent with its role as a binder requiring good dispersibility. Aerosil^®^200 presented an ultrafine particle size (0.012 µm, according to the manufacturer), which explains its high surface area and efficiency as a lubricant, despite the absence of measurable flow parameters.

Particle size distributions further indicate that calcium carbonate has the finest particles, whereas sodium naproxen and cellulose have broader distributions, which may influence powder compressibility, blend uniformity, and tablet homogeneity. Collectively, these data provide a foundation for predicting the handling behavior, mixing efficiency, and compaction performance of the powder blends during tablet production.

### 3.2. Design of Experiment

A face-centered central composite design (FCCCD, α = 1) consisting of 17 experimental runs, including three center points, was employed to investigate the effects of mixing time, fill level, and rotational speed on the properties of the powder blends and the resulting tablets. The total number of runs in FCCCD is given by Equation (1):

N = 2^k^ + 2k + 3 + C0, (1)

where *k* is the number of factors and *C*0 the number of center points. For three factors, mixing time (5, 12.5, 20 min), fill level (25%, 40%, 55%), and rotational speed (10, 20, 30 rpm) and three center points, the total number of experimental runs is *N* = 17. The DoE and response surface methodology analyses were performed using MATLAB Online.

Factor levels were selected based on prior knowledge: mixing time affects blend homogeneity; fill level influences granulation and flow; rotational speed controls shear forces impacting particle size and tablet mechanical properties.

Responses measured included angle of repose, angle of fall, angle of difference, average tablet thickness and mass, API content, and tablet abrasiveness, all reported with standard deviations to account for measurement variability.

This FCCCD enables efficient estimation of main, interaction, and quadratic effects while providing a reliable assessment of process reproducibility.

Analysis of the results demonstrates that powder flow properties were influenced differently by each factor (Table 2). The angle of repose ranged from 49.2° to 53.6°, with the highest values observed at longer mixing times combined with high rotational speeds, suggesting increased cohesiveness under these conditions. Lower fill levels tended to slightly reduce the angle of repose, improving flowability. The angle of fall and angle of difference followed similar trends, indicating that both the packing of the powder and the interparticle interactions were affected by the processing conditions. Notably, combinations of high fill level and low mixing time produced higher angles of difference, reflecting less uniform powder distribution.

Tablet characteristics also showed clear dependencies on the process parameters. Average tablet thickness varied from 3.130 mm to 3.498 mm, with longer mixing times and higher fill levels generally producing thicker tablets due to improved homogeneity and powder consolidation. Tablet mass followed similar trends, increasing with fill level and mixing time. API content remained relatively consistent, with values ranging from 0.0419 g to 0.1333 g per tablet. Deviations in API content were most pronounced under conditions of low mixing time and high fill level, indicating local inhomogeneities in the blend. Tablet abrasiveness was particularly sensitive to fill level, with the highest abrasiveness observed at high fill levels combined with low mixing time, likely due to insufficient binder distribution and weaker interparticle bonding. Conversely, tablets produced under intermediate conditions or longer mixing times exhibited lower abrasiveness, demonstrating improved mechanical integrity.

The results indicate that mixing time is crucial for blend homogeneity, fill level significantly influences tablet mass, thickness, and abrasiveness, and rotational speed affects powder cohesiveness and flow, particularly when combined with other factors. These findings provide a comprehensive understanding of the process–property relationships, offering guidance for optimizing manufacturing conditions to ensure consistent and reproducible tablet performance.

### 3.3. Blend Properties

The response surface analysis revealed distinct effects of mixing time, fill level, and rotational speed on powder flowability parameters.

**Figure 1:**
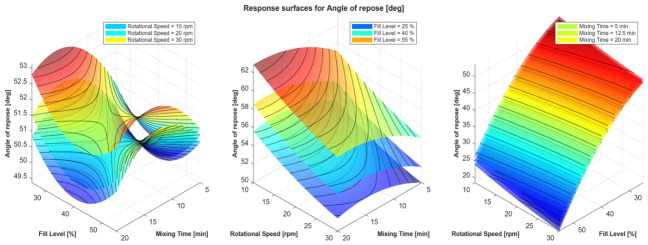
Response surface plots showing the effect of process variables on angle of repose. The colored surfaces correspond to the three levels of the third process variable indicated in the legend (rotational speed, fill level, or mixing time, depending on the plot). The surface color gradient represents the predicted response value, with blue indicating lower values and red indicating higher values.

For the angle of repose (Figure 1), values ranged from approximately 49° to 54°. The response surfaces indicate that the angle of repose was affected by the combined action of mixing time, fill level, and rotational speed. In general, higher rotational speeds were associated with lower angle values, suggesting improved flowability of the powder blend. The effects of mixing time and fill level were less pronounced and depended on the simultaneous values of the remaining process variables.

**Figure 2:**
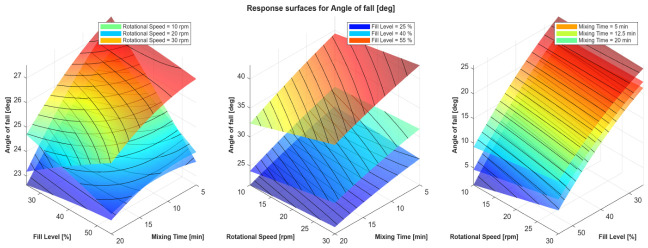
Response surface plots showing the effect of process variables on angle of fall. The colored surfaces correspond to the three levels of the third process variable indicated in the legend (rotational speed, fill level, or mixing time, depending on the plot). The surface color gradient represents the predicted response value, with blue indicating lower values and red indicating higher values.

In the case of the angle of fall (Figure 2), values varied between 22° and 28°. The dominant effect was exerted by fill level, where higher values promoted more efficient particle rearrangement and improved packing. Short mixing times, particularly at low fill levels, produced elevated angles of fall, signifying poorer particle mobility and reduced uniformity of the blends.

**Figure 3:**
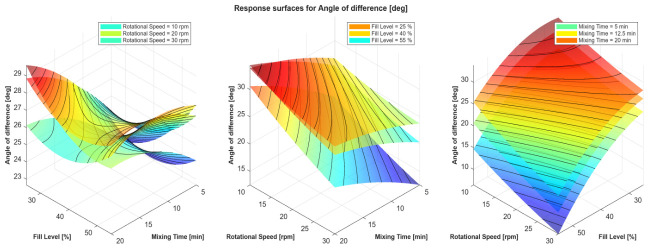
Response surface plots showing the effect of process variables on angle of difference. The colored surfaces correspond to the three levels of the third process variable indicated in the legend (rotational speed, fill level, or mixing time, depending on the plot). The surface color gradient represents the predicted response value, with blue indicating lower values and red indicating higher values.

Regarding the angle of difference (Figure 3), the results showed the highest sensitivity to process variations, with values spanning 22° to 29°. Maximal differences occurred under conditions of high mixing time combined with low fill level, suggesting inadequate powder distribution and heterogeneity in flow behavior. Conversely, intermediate parameter settings produced lower angle differences, indicative of more stable and reproducible flow characteristics.

These findings confirm that process variables significantly influence blend properties. Moderate conditions ensure balanced flowability, while extreme parameter combinations, particularly long mixing times with low fill or high rotational speed tend to increase cohesiveness and variability, which may compromise downstream processing efficiency.

### 3.4. Tablet Properties

The response surface methodology demonstrated that tablet characteristics were strongly influenced by the studied process variables, with distinct trends observed for thickness, mass, API content, and abrasiveness.

**Figure 4:**
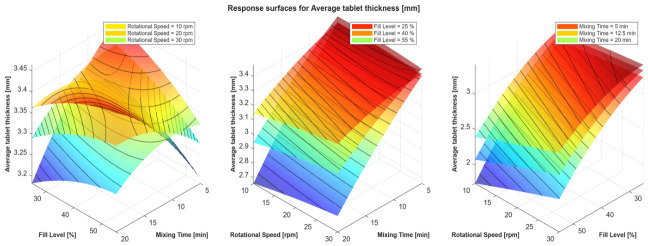
Response surface plots showing the effect of process variables on average tablet thickness. The colored surfaces correspond to the three levels of the third process variable indicated in the legend (rotational speed, fill level, or mixing time, depending on the plot). The surface color gradient represents the predicted response value, with blue indicating lower values and red indicating higher values.

It should be emphasized that the aim of this study was not to optimize the formulation of tablets with a constant weight, but to investigate the effect of technological parameters across a wide range of operating conditions. Therefore, variations in API content and tablet mass were intentionally allowed, which made it possible to more clearly identify the relationship between the process and the properties. With respect to tablet thickness (Figure 4), the values ranged from approximately 3.13 mm to 3.50 mm. The response surfaces indicate that tablet thickness was influenced by the combined effects of fill level, mixing time, and rotational speed. In general, higher fill levels tended to produce thicker tablets, while lower fill levels were associated with thinner compacts; however, these trends were dependent on the simultaneous values of the other process parameters, particularly rotational speed. Similarly, the effect of mixing time was not independent and varied across different levels of rotational speed and fill level. Overall, the observed variations in tablet thickness suggest a moderate sensitivity of the compaction process to the investigated mixing conditions.

**Figure 5:**
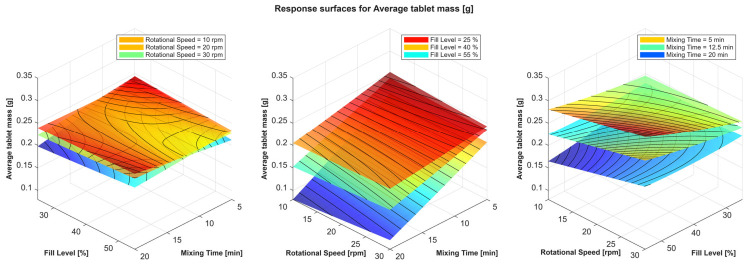
Response surface plots showing the effect of process variables on average tablet mass. The colored surfaces correspond to the three levels of the third process variable indicated in the legend (rotational speed, fill level, or mixing time, depending on the plot). The surface color gradient represents the predicted response value, with blue indicating lower values and red indicating higher values.

For tablet mass (Figure 5), variations followed a similar pattern, spanning from 0.192 g to 0.253 g. Among the investigated factors, fill level appeared to have a notable influence, with higher levels generally associated with increased mass. Mixing time also contributed to the observed variability, although its effect was more moderate and depended on interactions with other process parameters. Conditions corresponding to intermediate mixing times tended to support a relatively stable mass distribution, whereas more extreme process settings (very low or very high shear) were associated with a greater variability, which may indicate irregular die filling and possible segregation effects.

**Figure 6:**
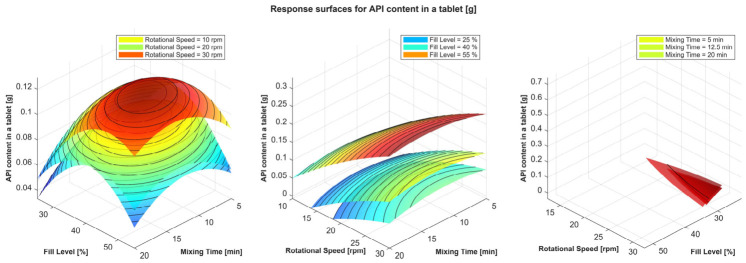
Response surface plots showing the effect of process variables on API content in a tablet. The colored surfaces correspond to the three levels of the third process variable indicated in the legend (rotational speed, fill level, or mixing time, depending on the plot). The surface color gradient represents the predicted response value, with blue indicating lower values and red indicating higher values.

**Figure 7:**
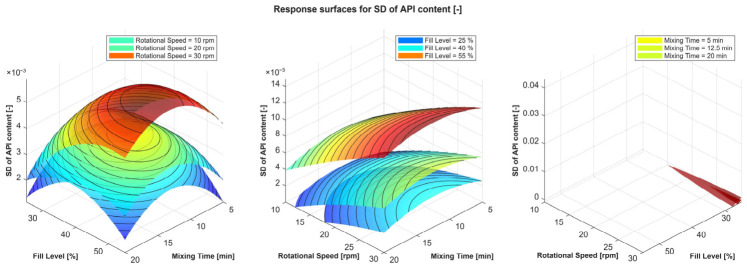
Response surface plots showing the effect of process variables on SD of API content in a tablet. The colored surfaces correspond to the three levels of the third process variable indicated in the legend (rotational speed, fill level, or mixing time, depending on the plot). The surface color gradient represents the predicted response value, with blue indicating lower values and red indicating higher values.

Concerning API content uniformity (Figure 6), the values per tablet ranged from 0.0419 g to 0.1333 g. The most stable SD of API distribution was obtained under intermediate mixing times and rotational speeds, conditions that ensured sufficient blending without promoting segregation (Figure 7). In contrast, short mixing times at high fill levels produced marked deviations, pointing to incomplete dispersion of the active ingredient within the excipient matrix.

**Figure 8:**
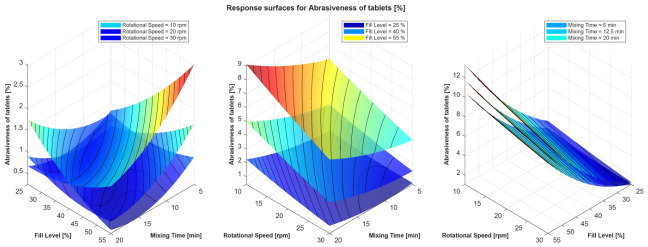
Response surface plots showing the effect of process variables on abrasiveness of tablets. The colored surfaces correspond to the three levels of the third process variable indicated in the legend (rotational speed, fill level, or mixing time, depending on the plot). The surface color gradient represents the predicted response value, with blue indicating lower values and red indicating higher values.

Tablet abrasiveness (Figure 8) exhibited the widest variability, with values spanning 0.3% to 3.7%. The most critical determinant was fill level: high fill levels in combination with short mixing times resulted in highly fragile tablets, likely due to inadequate binder distribution and insufficient interparticle bonding. Conversely, intermediate process conditions minimized abrasiveness, yielding mechanically more robust compacts suitable for further handling.

Overall, these results highlight that process variables must be carefully balanced to achieve desirable tablet properties. While increased fill levels enhance mass and thickness, they simultaneously pose risks for API inhomogeneity and reduced mechanical strength if not coupled with adequate mixing. The FCCCD approach thus provided valuable insights into parameter optimization to ensure both uniformity and robustness of the final dosage form.

### 3.5. Response Surface Analysis of Tablet Properties

The relationships between the three process variables—mixing time (MT, min), fill level (FL, %), and rotational speed (RS, rpm)—and seven critical tablet properties were modeled using second-order polynomial regression. Each dependent variable *Y* is described by Equation (2):

(2) $$Y=\left(\beta_{0}+\varepsilon \right)+\beta_{MT}MT+\beta_{FL}FL{+\beta }_{RS}RS+\beta_{MM}{MT}^{2}+\beta_{FF}{FL}^{2}+\beta_{RR}{RS}^{2}+\beta_{MF}MTFL+\beta_{MR}MTRS\;+\beta_{FR}FLRS$$

where *β*_0_ is the intercept, *β_MT_*, *β_FL_*, *β_RS_*, represent linear effects of each process variable, *β_MT_*_2_, *β_FL_*_2_, *β_RS_*_2_ represent quadratic (nonlinear) effects, *β_MF_*, *β_MR_*, *β_FR_* are interaction terms, and *ε* is the residual error.

The angle of repose quantifies powder flowability (Equation (3)). Linear coefficients indicate that increasing mixing time enhances flowability, whereas higher fill level and rotational speed reduce it slightly. Quadratic and interaction terms capture subtle curvature and combined effects. Surface plots reveal that at low rotational speeds (10 rpm), the angle increases with longer mixing times and moderate fill levels, while high rotational speeds (30 rpm) lower the overall angle. Contours indicate that Fill Level has a nonlinear effect, strongest at intermediate levels.

(3) $$\begin{array}{l} \mathrm{Angle of repose}\\ \;\;\;\;\;=54.41303+0.50870\;MT-0.33830\;FL-0.08497\;RS-0.01827\;{MT}^{2}-0.00200\;MTFL\;\\ \;\;\;\;\;+\;0.00400\;MTRS+0.00477\;{FL}^{2}-0.00100\;FLRS+0.00322\;{RS}^{2} \end{array}$$

The angle of fall reflects powder settling behavior and cohesion (Equation (4)). Rotational speed has the strongest negative effect, decreasing the angle as speed increases. Surface plots show that at higher fill levels, the angle slightly increases, while longer mixing times marginally reduce it. Interaction terms manifest as slight tilts in the surfaces, showing that the combined influence of mixing and rotation modifies the cohesion.

(4) $$\begin{array}{l} \mathrm{Angle of fall}=36.94831-0.16190\;MT-0.20328\;FL-0.65814\;RS-0.00536\;{MT}^{2}-0.00600\;MTFL\\ \;\;\;\;\;+\;0.00433\;MTRS+0.00023\;{FL}^{2}-0.00600\;FLRS+0.01298\;{RS}^{2} \end{array}$$

The angle of difference measures powder cohesiveness (Equation (5)). Surface plots demonstrate that longer mixing times and higher rotational speeds increase cohesion. Fill Level has a minor nonlinear effect, observable as a gentle curvature in the surfaces. Interaction terms indicate that the effect of one variable is slightly modulated by the level of the other, producing subtle tilts and curvatures on the surface.

(5) $$\begin{array}{l} \mathrm{Angle of difference}\\ \;\;\;\;\;=17.46471+0.67061MT\;-0.13503\;FL+0.57318\;RS-0.01290\;{MT}^{2}-0.00800\;MTFL\\ \;\;\;\;\;+\;0.00833\;MTRS+0.00500\;{FL}^{2}-0.00700\;FLRS-0.00976\;{RS}^{2} \end{array}$$

Tablet thickness is moderately influenced by rotational speed and marginally by mixing time (Equation (6)). Surface plots indicate an overall increase in thickness with higher rotational speeds. Contours remain relatively parallel, suggesting limited nonlinear interactions, although small curvatures show that extreme combinations of mixing time and rotation produce slight deviations from linearity.

(6) $$\begin{array}{l} \mathrm{Average tablet thickness}=3.09852+0.01599\;MT+0.00012\;FL+0.02046\;RS-0.00054\;{MT}^{2}+\\ \;\;\;0.00048\;MTFL-0.00107\;MTRS-0.00008\;{FL}^{2}-0.00002\;FLRS-0.00019\;{RS}^{2} \end{array}$$

Tablet mass reflects powder quantity per tablet (Equation (7)). Linear coefficients are small, but surface plots reveal that rotational speed slightly increases mass, whereas mixing time and fill level reduce it slightly. Quadratic and interaction terms introduce gentle curvatures, visible as small bends in the surface contours.

(7) $$\begin{array}{l} \mathrm{Average tablet mass}\\ \;\;\;\;\;=0.25137-0.00035\;MT-0.00174\;FL+0.00231\;RS+0.00003\;{MT}^{2}+0.00007\;MTFL\\ \;\;\;\;\;-\;0.00017\;MTRS-0.00001\;{FL}^{2}+0.00002\;FLRS-0.00003\;{RS}^{2} \end{array}$$

API content ensures dosage uniformity (Equation (8)). Surface plots indicate that fill level and mixing time slightly increase API content, while higher rotational speeds have a minor reducing effect. Contours and gradient colors illustrate that these effects are additive and moderately linear, with slight nonlinearities arising from interaction terms.

(8) $$\begin{array}{l} \mathrm{API content}=-0.04801+0.00506\;MT+0.00985\;FL-0.00858\;RS-0.00038\;{MT}^{2}+0.00006\;MTFL+\\ \;\;\;\;\;\;\;0.00007\;MTRS-0.00015\;{FL}^{2}+0.00010\;FLRS+0.000012\;{\;RS}^{2} \end{array}$$

The abrasive properties of the powder system increase mechanical wear of tablet press tooling and related process equipment. (Equation (9)). Surface plots show a strong negative effect of mixing time, reducing abrasiveness, while fill level increases it. Rotational speed moderately reduces abrasiveness. Contours highlight that interactions, especially between mixing time and rotational speed, produce curved regions, indicating complex combined effects of process parameters on tablet abrasiveness.

(9) $$\begin{array}{l} \mathrm{Abrasiveness}=4.86314-0.87298\;MT+0.60340\;FL-0.56090\;RS+0.04989\;{MT}^{2}-0.03397\;MTFL\\ \;\;\;\;\;+\;0.03754\;MTRS+0.00803\;{FL}^{2}-0.03455\;FLRS+0.03018\;{\;RS}^{2} \end{array}$$

Across all response surfaces, rotational speed generally shifts the system response, often in the negative direction for parameters associated with powder flow behaviour and cohesiveness.

### 3.6. Statistical Analysis

Across all response surfaces, rotational speed generally shifts the system response, often in the negative direction for parameters associated with powder flow behaviour and cohesiveness. Mixing time tends to increase the angle of repose and angle of difference, while fill level exhibits non-linear effects that are most pronounced at intermediate values. The contour patterns observed on the response surfaces reflect these interactions and nonlinearities, highlighting the usefulness of response surface methodology for analysing process behaviour and factor interplay.

A quadratic model (RSM) was fitted for all responses, and the quality of fit, evaluated in terms of R^2^, adjusted R^2^, and RMSE (Table 3), varied considerably between variables. The model provided the most reliable description for the angle of difference, whereas substantially lower predictive capability was observed for API content. Intermediate model performance was obtained for tablet mass, thickness, and abrasiveness, indicating partial sensitivity of these responses to process conditions. In contrast, the relatively weak model fit for angle of repose and angle of fall suggests that these parameters are less influenced by processing variables and are primarily governed by material properties.

The statistical significance of regression coefficients revealed that the influence of process parameters was highly response-dependent. For the angle of repose and angle of fall, none of the model terms were statistically significant (*p* > 0.05), indicating that these parameters were primarily governed by material properties. In contrast, the angle of difference showed statistically significant contributions from both linear terms (mixing time and rotational speed) and interaction effects, confirming its sensitivity to process conditions. For tablet properties, selected interaction terms (notably mixing time–rotational speed) were found to be significant for both tablet mass and thickness, suggesting that these responses are governed by combined process effects rather than individual factors. No statistically significant regression terms were observed for API content, which may reflect the inherently high variability of this response due to segregation phenomena. The relatively low adjusted R^2^ values observed for selected responses indicate that a significant portion of variability is not captured by the model, likely due to complex phenomena such as stochastic powder behaviour.

One-way ANOVA was performed to quantify the effect of individual process variables: mixing time, fill level, and rotational speed, on blend and tablet properties.

**Figure 9:**
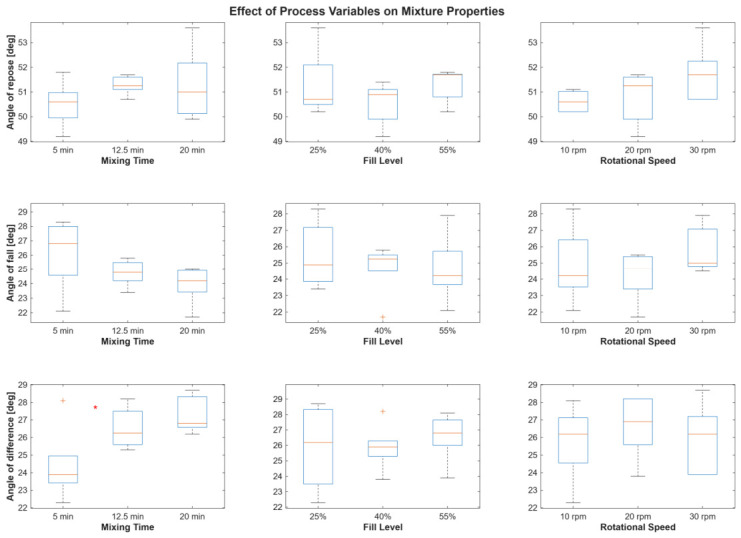
Boxplots illustrating the influence of process variables on powder mixture properties. Asterisks indicate levels of statistical significance based on one-way ANOVA. The plus signs indicate an outlier.

For powder mixture properties (Table 4, Figure 9), no statistically significant influence of the studied factors was observed for the angle of repose or angle of fall (*p* > 0.05), indicating limited sensitivity of these parameters to process conditions. Mean values for the angle of repose remained consistent across all categories (≈50–52°), while the angle of fall showed a slight decrease at longer mixing times (≈23°) compared with shorter mixing durations (≈26°). In contrast, a significant effect of mixing time was identified for the angle of difference (*p* = 0.0245), indicating increased sensitivity of this parameter to processing conditions.

**Figure 10:**
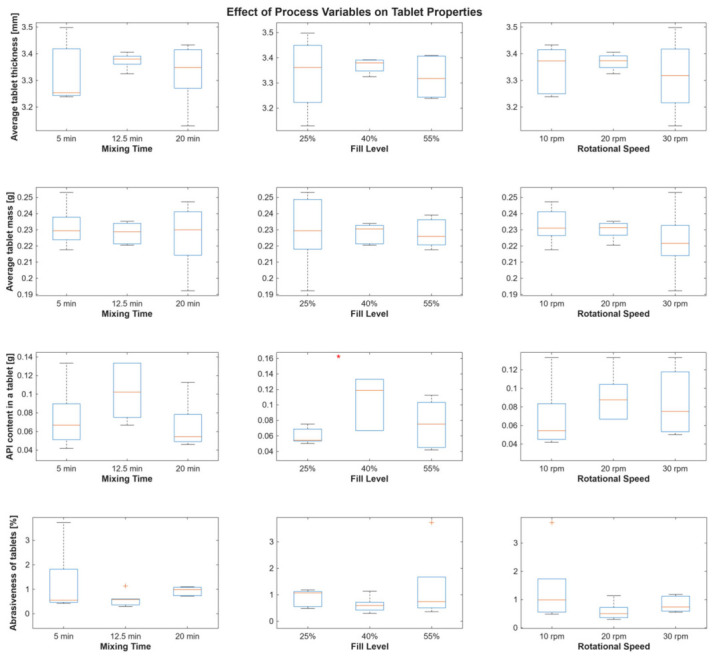
Boxplots illustrating the influence of process variables on tablet properties. Asterisks indicate levels of statistical significance based on one-way ANOVA. The plus signs indicate an outlier.

In the case of tablet properties (Table 5, Figure 10), most responses were not significantly affected by the process variables. Average thickness and mass remained consistent across all conditions (*p* > 0.05), supporting the limited sensitivity observed in the regression analysis. API content, however, was significantly influenced by fill level (*p* = 0.0411), with the highest values observed at intermediate fill levels (40%), likely reflecting segregation effects within the blend. Tablet abrasiveness exhibited substantial variability, although differences did not reach statistical significance. Nevertheless, a tendency toward higher abrasiveness at high fill levels and low rotational speed suggests reduced binder distribution and weaker interparticle bonding.

Overall, the ANOVA results confirm that most process parameters exert limited effects within the investigated range, while highlighting mixing time and fill level as the most relevant factors. These observations are consistent with the response surface analysis and support the identification of key process variables governing system behaviour. The results obtained from ANOVA are consistent with the response surface modelling, although the two approaches provide complementary perspectives. While ANOVA identifies differences between discrete factor levels, the RSM analysis further reveals interaction effects and nonlinear behaviour, which are not captured in one-way analysis.

In particular, selected responses (e.g., tablet mass and thickness) exhibited significant interaction effects in the regression models despite non-significant main effects in ANOVA, highlighting the importance of combined process conditions. In the case of API content, the discrepancy between ANOVA and RSM suggests increased variability and limited model predictability, likely associated with segregation phenomena. 

### 3.7. The Optimization of Process

The optimization of pharmaceutical powder blending processes requires a thorough understanding of the relationships between process parameters and critical quality attributes (CQAs). In this study, a systematic design space analysis was conducted to evaluate the effects of mixing time (MT), fill level (FL), and rotational speed (RS) on key product characteristics, including active pharmaceutical ingredient (API) content, angle of difference, and abrasiveness. Quadratic response surface models (RSM) were developed based on experimental central composite design data, enabling the prediction of process outcomes across a wide range of operating conditions.

Acceptance criteria were defined a priori based on technological performance requirements. Adequate blend homogeneity was ensured by requiring the predicted angle of difference to remain below 27°. API content was constrained to lie within ±5% of the experimental mean API value, reflecting acceptable assay variability. Abrasiveness values were limited to a maximum of 1.0 to avoid excessive mechanical degradation of the material. Only operating conditions simultaneously fulfilling all three criteria were considered acceptable for further analysis.

To enable ranking and visualization of acceptable operating conditions, individual desirability functions were defined for each response variable. Linear desirability functions with hard cutoffs were employed. These functions map predicted responses onto a dimensionless scale between 0 (unacceptable) and 1 (fully desirable). The formulation was intentionally kept simple and transparent and does not represent the classical Harrington log–exponential desirability function.

API content was treated as a target response, with maximum desirability assigned to the experimental mean API value. Desirability decreased linearly with increasing deviation from the target and reached zero at ±5% deviation (Equation (10)):

(10) $$d_{API}\left(y\right)=max\left(0.1-\frac{\left|y-\bar{API}\right|}{0.05\bar{API}}\right)$$

For angle of difference, a minimization-type desirability function was applied by Equation (11):

(11) $$d_{Angle\;of\;difference}\left(y\right)=max\left(0,\;min\left(1,\frac{27-y}{27}\right)\right)$$

Predicted values exceeding 27° were assigned zero desirability. A similar linear desirability function was used for abrasiveness (Equation (12)):

(12) $$d_{Abrasiveness}\left(y\right)=max\left(0,min\left(1,\frac{1.0-y}{1.0}\right)\right)$$

The overall desirability was calculated as the unweighted product of the individual desirability functions (Equation (13)):

(13) $$D\left(MT,FL,RS\right)=d_{API}\cdot d_{Angle\;of\;difference}\cdot d_{Abrasiveness}$$

All responses were assigned equal importance. Operating points that failed to meet at least one acceptance criterion were assigned an overall desirability of zero.

The three-dimensional operational design space was defined as the set of all combinations of mixing time, fill level, and rotational speed for which all acceptance criteria were satisfied. The desirability function was used exclusively to rank and visualize acceptable operating points within this region and did not influence the shape or boundaries of the operational window.

**Figure 11:**
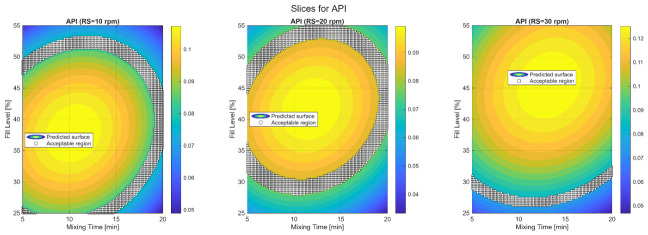
Contour plot showing acceptable regions of API content of tablets.

**Figure 12:**
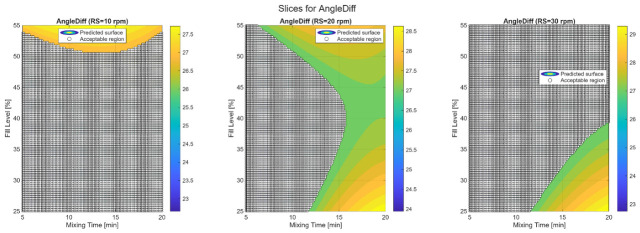
Contour plot showing acceptable regions of particle angle of difference.

**Figure 13:**
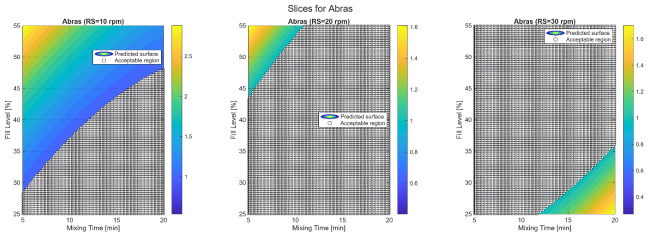
Contour plot showing acceptable regions of abrasiveness of tablets.

The contour plots representing the design space at discrete rotational speeds of 10, 20, and 30 rpm (Figure 11, Figure 12 and Figure 13) illustrate the influence of process parameters on compliance with technological criteria. At a rotational speed of 10 rpm, the acceptable operational region is concentrated at lower mixing times, approximately 5–12 min, and fill levels between 25% and 40%, indicating that reduced mechanical energy requires tighter control of mixing duration and fill volume to maintain uniformity and prevent excessive abrasion. At 20 rpm, the acceptable region expands toward longer mixing times of 8–18 min and fill levels of 30–50%, reflecting the increased mixing efficiency that permits a wider range of operational conditions while still satisfying all quality criteria. At the highest rotational speed of 30 rpm, the contour plot reveals that acceptable conditions are predominantly located at intermediate mixing times of 12–20 min and higher fill levels of 35–55%, demonstrating that elevated rotational speed shifts the operational window toward longer mixing times and larger batch volumes, while maintaining compliance with technological specifications. Across all three levels of rotational speed, the contour plots indicate that the interplay between mixing time and fill level is critical, and that careful selection of these parameters is required to ensure consistent API content, controlled particle uniformity, and minimal abrasiveness. These findings provide practical guidance for process design and optimization, identifying precise operational windows at each rotational speed that enable robust production and regulatory compliance.

**Figure 14:**
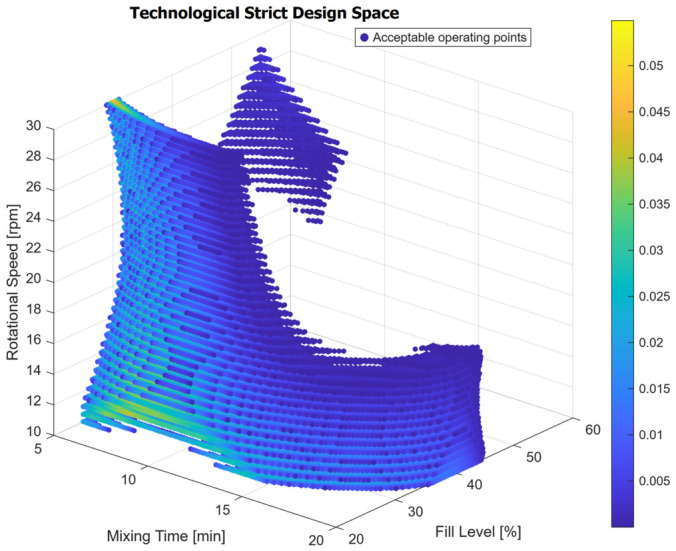
Three-dimensional representation of the design space, illustrating all combinations of mixing time, fill level, and rotational speed that comply with technological criteria.

The three-dimensional design space (Figure 14) for the powder mixing process, defined according to technological criteria of API content (±5% of the mean), particle angle difference (≤27°), and abrasiveness (≤1%), exhibits a complex and non-uniform geometry. Two distinct regions of acceptable operation are observed, corresponding to separate combinations of process parameters that satisfy all quality requirements. The lower region is associated with a rotational speed of approximately 10–15 rpm, mixing times between 5 and 12 min, and fill levels ranging from 25% to 40%. The upper region corresponds to higher rotational speeds of 20–30 rpm, mixing times of 12–20 min, and similar fill levels of 35–55%. These findings indicate that compliance with technological specifications is governed by the interaction between all three process variables, rather than by any single factor independently. The partially transparent surfaces in the visualization represent the isosurface of acceptable points; variations in apparent intensity are attributable to the superposition of surface layers and the applied lighting, rather than differences in the density of acceptable points. From a practical perspective, the identified regions define operational windows that ensure consistent API content, maintain particle uniformity, and prevent excessive abrasion. Process operation within the specified ranges, specifically a mixing time of 8–18 min, fill levels of 30–50%, and rotational speeds of 10–30 rpm, provides a robust and flexible framework for maintaining product quality while accommodating variations in process conditions. These results provide clear guidance for process optimization and control, facilitating the selection of parameter sets that maximize product consistency and compliance with regulatory standards.

All experiments were performed using small tablets with a diameter of 6 mm. This tablet size was deliberately selected to ensure sufficient mechanical sensitivity and to allow subsequent extrapolation of the obtained operational design space toward larger tablet sizes. The applicability of the identified operating window to smaller tablets is not expected, due to fundamentally different stress transmission and contact mechanics at reduced dimensions.

## 4. Discussion

The present study indicates that the behaviour of the sodium naproxen–calcium carbonate formulation is governed primarily by the interplay between mixing time and fill level, rather than by a strong main effect of rotational speed within the tested operating window. In particular, the angle of difference was the only powder-flow descriptor that showed a statistically significant dependence on mixing time, whereas fill level was the only factor that significantly affected API content. The increase in angle of difference observed at longer mixing times may indicate the formation of stronger interparticle interactions within the blend. Prolonged exposure to shear forces can promote particle rearrangement and the development of cohesive agglomerates, resulting in reduced powder mobility after vibration. The relatively narrow range of angle of repose values observed throughout the experimental design suggests that the flow properties of the blends were dominated by the excipient composition rather than by the investigated mixing parameters. The presence of microcrystalline cellulose and colloidal silica likely contributed to maintaining acceptable flowability across the studied processing window.

The significant influence of fill level on API content may be related to differences in powder circulation patterns within the mixing vessel. At higher fill levels, particle movement becomes more restricted, which may affect the distribution of sodium naproxen within the blend and consequently influence content uniformity.

Tablet thickness and mass remained comparatively stable across the experimental domain, suggesting that the formulation preserved acceptable compressibility under the conditions studied. Tablet abrasiveness showed the widest numerical variability and tended to increase under high fill levels combined with short mixing times, which is consistent with insufficient homogenization and less effective binder distribution. Higher abrasiveness values observed under selected processing conditions may result from reduced tablet densification and weaker interparticle bonding. Insufficient blend homogenization may lead to local variations in particle packing, producing tablets that are more susceptible to mechanical damage during handling. These findings are in line with the broader literature indicating that blend uniformity in direct compression is highly sensitive to cohesive behaviour and to the mechanical history of the powder bed. Reviews on continuous direct compression and powder segregation emphasize that losses in content uniformity often originate during blend transfer, hopper discharge, and die filling rather than from a single formulation variable alone [1,26]. The present results, particularly the significant influence of fill level on API content, support this interpretation and suggest that blender loading should be treated as a critical process variable when cohesive materials are processed by direct compression [26].

The choice of mixing technology strongly influenced the homogeneity and quality of sodium naproxen tablets. High-energy or vibratory mixing can improved blend homogeneity and API distribution relative to low-energy tumbling, whereas excessive mechanical input could introduce local over-processing and variability in tablet properties [9]. This aligns well with the present work, in which the tested blending window produced only modest changes in the simple flow indices but still affected blend cohesiveness and dosage uniformity in a process-dependent manner.

Sodium naproxen typically constitutes a cohesive active pharmaceutical ingredient, often characterized by relatively fine particles and strong interparticle interactions, which may promote agglomeration and segregation in powder blends [9]. Such cohesive behaviour can lead to increased variability in API distribution and limit the predictability of process outcomes, particularly under low-energy mixing or uneven powder flow conditions [9]. This may explain the relatively low model predictability and the discrepancy between ANOVA and RSM results observed for API content in the present study, suggesting that segregation phenomena play a significant role in governing this response.

Microcrystalline cellulose (MCC) exhibits predominantly plastic deformation during compaction, which promotes the formation of strong interparticle bonds through hydrogen bonding between plastically deformed particles. This behaviour is widely recognised as a key factor responsible for its excellent compactibility and binding properties [27,28]. At the same time, MCC is known to display relatively high cohesiveness and limited inherent flowability, particularly in finer grades, which can lead to increased sensitivity to mixing conditions and particle interactions within the blend [28]. Consequently, the presence of MCC in the formulation may contribute to the observed trends in flow-related parameters and the development of interparticle cohesion, especially under prolonged mixing or higher shear conditions.

Calcium carbonate is typically described as a brittle excipient; however, its bulk behaviour is strongly influenced by particle size, morphology, and surface characteristics. In particular, powders composed of irregular or fine particles can exhibit increased interparticle friction and cohesion, leading to reduced flowability [29]. More generally, powder cohesion, arising from interparticle forces such as van der Waals interactions, surface roughness, and agglomeration, represents a major limitation for powder flow and processing in pharmaceutical systems [1]. In the present study, the observed limited flowability of calcium carbonate is therefore consistent with its particle characteristics and supports its selection as a challenging model material for investigating process–property relationships. The combined presence of cohesive MCC and physically heterogeneous calcium carbonate may therefore lead to competing mechanisms of cohesion and fragmentation, which manifest as non-linear and interaction-driven responses observed in the experimental design.

From a Quality by Design perspective, the results support a conservative interpretation of the operating space rather than a single universal optimum. Yeom and Choi demonstrated, using a QbD–DEM framework, that the effective blending window may shift after scale-up and that pilot-scale conditions can require shorter blending times than laboratory-scale settings to meet target values [20]. Similarly, process-modelling studies in Pharmaceutics have shown that direct compression requires adequate flow, blend uniformity, compaction behaviour, and ejection properties, and that tablet weight variability can remain sensitive to process assumptions even when the formulation appears robust at bench scale [30]. In the present study, the two acceptable regions identified in the design space indicate that multiple combinations of mixing time, fill level, and rotational speed can satisfy the selected acceptance criteria, but only within a limited and non-linear operating window.

Importantly, the present data also indicate that improved blend homogeneity does not automatically translate into lower abrasiveness. This is an important practical point because cohesive powders may respond favourably in terms of content distribution while still showing signs of mechanical degradation under overly aggressive processing conditions. Such trade-offs are well recognised in powder engineering and support the use of a QbD-based strategy in which flowability, content uniformity, and mechanical integrity are evaluated together rather than independently [1,6,14,15,26]. Within that framework, the present study suggests that mixing time primarily contributes to blend stabilisation, whereas fill level exerts a stronger influence on dose distribution. The resulting process window may therefore be useful for balancing homogeneity and robustness in cohesive direct-compression formulations.

Overall, the results extend previous work on sodium naproxen direct-compression systems by showing that, for this formulation, the most relevant optimisation lever is not a single mixer setting but the combined selection of mixing time and fill level. The data further support the view that cohesive systems require process development strategies that are formulation-specific and scale-aware, especially when the goal is to maintain both dosage uniformity and acceptable mechanical performance [9,26,30].

## 5. Conclusions

The results demonstrated that process parameters exert highly selective and property-specific effects rather than uniform influences across all critical quality attributes. In particular, mixing time was identified as the dominant factor governing blend homogeneity, as reflected by the angle of difference, while fill level primarily controlled active pharmaceutical ingredient content uniformity. Rotational speed showed secondary but non-linear effects, particularly in combination with other variables, influencing both flow behaviour and tablet mechanical performance.

Importantly, the application of response surface methodology enabled the definition of a modelled design space for direct compression of a highly cohesive powder system. Two distinct operational regions satisfying all predefined quality criteria were identified, demonstrating that acceptable product quality can be achieved through different combinations of process parameters rather than a single optimal point.

These findings fill an important gap between empirical powder blending studies and systematic process design approaches. They provide a mechanistic understanding of how mixing energy distribution affects cohesion-driven segregation and consolidation phenomena in direct compression. From a practical perspective, the results support the implementation of Quality by Design principles in the development and scale-up of cohesive powder formulations, enabling more predictable manufacturing of solid dosage forms.

## Figures and Tables

**Table 1:** Properties of pharmaceutical ingredients.

Sample	Angle of Repose	Angle of Fall	Angle of Difference	d(0.5)	d(0.9)	d(0.1)
	**[deg]**	**[deg]**	**[deg]**	**[um]**	**[um]**	**[um]**
Sodium naproxen	45.9	23.4	22.5	154	258	17
Polyvinylpyrrolidone (PVP)	39.6	16.5	23.1	21	50	4
Cellulose	44.6	23.2	21.4	123	178	11
Calcium carbonate	42.8	29.5	13.3	6	10	0.7
Aerosil^®^200	-	-	-	0.012 ^1^	-	-

^1^ According to the manufacturer.

**Table 2:** Effect of process parameters on the physical properties of blends and tablets.

Run	Mixing Time [min]	Fill Level [%]	Rotational Speed [rpm]	Angle of Repose [deg] ± SD	Angle of Fall [deg] ± SD	Angle of Difference [deg] ± SD	Average Tablet Thickness [mm] ± SD	Average Tablet Mass [g] ± SD	API Content in a Tablet [g] ± SD	Abrasiveness of Tablets [%] ± SD
1	5	25	10	50.6 ± 0.97	28.3 ± 0.37	22.3 ± 0.52	3.254 ± 0.113	0.229 ± 0.011	0.067 ± 0.001	0.485 ± 0.035
2	20	25	10	50.2 ± 0.64	24.0 ± 0.27	26.2 ± 0.56	3.433 ± 0.210	0.247 ± 0.023	0.054 ± 0.003	1.072 ± 0.099
3	5	55	10	50.2 ± 0.90	22.1 ± 0.39	28.1 ± 0.67	3.239 ± 0.201	0.218 ± 0.016	0.042 ± 0.001	3.727 ± 0.244
4	20	55	10	51.0 ± 0.63	24.2 ± 0.41	26.8 ± 0.71	3.409 ± 0.111	0.239 ± 0.014	0.046 ± 0.001	0.988 ± 0.089
5	5	25	30	50.7 ± 0.56	26.8 ± 0.28	23.9 ± 0.61	3.498 ± 0.076	0.253 ± 0.010	0.061 ± 0.003	1.185 ± 0.117
6	20	25	30	53.6 ± 0.74	24.9 ± 0.28	28.7 ± 0.74	3.130 ± 0.131	0.192 ± 0.058	0.050 ± 0.002	1.101 ± 0.090
7	5	55	30	51.8 ± 0.58	27.9 ± 0.47	23.9 ± 0.52	3.245 ± 0.057	0.226 ± 0.013	0.075 ± 0.003	0.556 ± 0.054
8	20	55	30	51.7 ± 0.54	25.0 ± 0.44	26.7 ± 0.59	3.318 ± 0.039	0.222 ± 0.005	0.113 ± 0.006	0.741 ± 0.037
9	12.5	40	20	51.1 ± 0.61	25.5 ± 0.39	25.6 ± 0.61	3.325 ± 0.097	0.220 ± 0.011	0.104 ± 0.005	1.137 ± 0.110
10	12.5	25	20	51.6 ± 0.90	23.4 ± 0.33	28.2 ± 0.57	3.361 ± 0.087	0.227 ± 0.009	0.073 ± 0.004	0.581 ± 0.037
11	12.5	55	20	51.7 ± 1.00	24.2 ± 0.29	27.5 ± 0.68	3.406 ± 0.067	0.235 ± 0.009	0.100 ± 0.004	0.362 ± 0.023
12	12.5	40	10	51.1 ± 0.52	25.8 ± 0.35	25.3 ± 0.57	3.373 ± 0.044	0.231 ± 0.005	0.132 ± 0.005	0.582 ± 0.051
13	12.5	40	30	50.7 ± 0.72	24.5 ± 0.45	26.2 ± 0.62	3.391 ± 0.067	0.221 ± 0.008	0.134 ± 0.006	0.604 ± 0.046
14	5	40	20	49.2 ± 0.80	25.4 ± 0.44	23.8 ± 0.67	3.392 ± 0.114	0.233 ± 0.012	0.131 ± 0.004	0.422 ± 0.024
15	20	40	20	49.9 ± 0.69	21.7 ± 0.36	28.2 ± 0.57	3.348 ± 0.124	0.230 ± 0.014	0.063 ± 0.002	0.721 ± 0.039
16	12.5	40	20	51.4 ± 0.71	25.1 ± 0.38	26.3 ± 0.65	3.387 ± 0.080	0.234 ± 0.009	0.070 ± 0.002	0.305 ± 0.025
17	12.5	40	20	51.7 ± 0.75	25.3 ± 0.40	26.7 ± 0.70	3.400 ± 0.085	0.242 ± 0.010	0.068 ± 0.003	0.310 ± 0.027

**Table 3:** Statistical parameters of the fitted response surface models.

Response	R^2^	Adj. R^2^	RMSE
Angle of repose	0.673	0.254	0.847
Angle of fall	0.639	0.176	1.573
Angle of difference	0.895	0.760	0.892
Average tablet thickness	0.721	0.363	0.0708
Average tablet mass	0.769	0.473	0.0099
API content	0.604	0.096	0.0297
Abrasiveness	0.706	0.327	0.650

**Table 4:** Results of one-way ANOVA showing the effect of process variables on powder mixture properties.

Mixture Properties								
Angle of repose [deg]								
Factor	F	*p*	Category	Mean	SD	Median	Min	Max
Mixing Time	1.033	0.3835	5 min	50.6	0.8	50.7	49.2	51.8
			12.5 min	51.2	0.28	51.1	51.1	51.7
			20 min	51.2	1.53	51	49.9	53.6
Fill Level	1.049	0.3781	25%	51.5	1.57	51.1	50.2	53.6
			40%	50.9	0.77	51.1	49.2	51.7
			55%	51.2	0.74	51	50.2	51.8
Rotational Speed	1.879	0.1919	10 rpm	50.7	0.36	50.7	50.2	51.1
			20 rpm	51.1	0.87	51.3	49.2	51.7
			30 rpm	51.7	1.05	51.7	50.7	53.6
Angle of fall [deg]								
Factor	F	*p*	Category	Mean	SD	Median	Min	Max
Mixing Time	2.11	0.1608	5 min	25.9	2.47	26.1	22.1	28.3
			12.5 min	24.9	0.91	25	23.4	25.8
			20 min	23.3	1.18	24.2	21.7	24.9
Fill Level	0.319	0.7325	25%	25.5	2.05	25	23.4	28.3
			40%	24.6	1.29	25	21.7	25.8
			55%	24.8	1.89	24.5	22.1	27.9
Rotational Speed	1.117	0.3567	10 rpm	24.6	2.09	25	21.7	28.3
			20 rpm	24.6	1.18	25	23.4	25.5
			30 rpm	25.4	1.32	25	24.5	27.9
Angle of difference [deg]								
Factor	F	*p*	Category	Mean	SD	Median	Min	Max
Mixing Time	4.999	0.0245 *	5 min	23.9	0.77	23.9	22.3	25.4
			12.5 min	26.8	1	26.3	25.3	28.2
			20 min	27.9	0.92	26.8	26.2	28.7
Fill Level	0.227	0.8003	25%	26.6	1.4	26.2	23.9	28.7
			40%	26.1	1.37	26.3	23.8	28.2
			55%	25.9	1.41	26.2	22.3	27.9
Rotational Speed	0.308	0.7399	10 rpm	25.8	2.14	26	22.3	28.3
			20 rpm	26	1.43	26.2	23.8	28.2
			30 rpm	26.4	1.44	26.2	23.9	28.7

* Statistically significant difference (*p* < 0.05).

**Table 5:** Results of one-way ANOVA showing the effect of process variables on tablet properties.

Tablet Properties								
Average tablet thickness [mm]								
Factor	F	*p*	Category	Mean	SD	Median	Min	Max
Mixing Time	0.475	0.6322	5 min	3.33	0.11	3.32	3.24	3.5
			12.5 min	3.37	0.03	3.38	3.33	3.41
			20 min	3.32	0.14	3.37	3.13	3.43
Fill Level	0.354	0.7082	25%	3.34	0.13	3.37	3.13	3.5
			40%	3.36	0.03	3.37	3.33	3.39
			55%	3.34	0.09	3.33	3.24	3.41
Rotational Speed	0.443	0.6517	10 rpm	3.35	0.09	3.37	3.24	3.41
			20 rpm	3.36	0.03	3.36	3.33	3.39
			30 rpm	3.32	0.14	3.33	3.13	3.5
Average tablet mass [g]								
Factor	F	*p*	Category	Mean	SD	Median	Min	Max
Mixing Time	0.202	0.8197	5 min	0.23	0.01	0.23	0.22	0.25
			12.5 min	0.23	0.01	0.23	0.22	0.24
			20 min	0.23	0.02	0.23	0.19	0.25
Fill Level	0.022	0.9779	25%	0.22	0.03	0.23	0.19	0.25
			40%	0.23	0.01	0.23	0.22	0.24
			55%	0.23	0.01	0.23	0.22	0.24
Rotational Speed	0.694	0.5172	10 rpm	0.23	0.01	0.23	0.22	0.24
			20 rpm	0.23	0.01	0.23	0.22	0.23
			30 rpm	0.23	0.02	0.23	0.19	0.25
API content in a tablet [g]								
Factor	F	*p*	Category	Mean	SD	Median	Min	Max
Mixing Time	2.198	0.1506	5 min	0.061	0.015	0.055	0.042	0.075
			12.5 min	0.093	0.029	0.071	0.067	0.133
			20 min	0.07	0.03	0.066	0.046	0.133
Fill Level	4.121	0.0411 *	25%	0.07	0.015	0.067	0.054	0.082
			40%	0.095	0.027	0.071	0.067	0.133
			55%	0.069	0.024	0.075	0.042	0.113
Rotational Speed	0.655	0.5356	10 rpm	0.084	0.036	0.071	0.042	0.133
			20 rpm	0.09	0.024	0.071	0.067	0.133
			30 rpm	0.064	0.02	0.055	0.046	0.113
Abrasiveness of tablets [%]								
Factor	F	*p*	Category	Mean	SD	Median	Min	Max
Mixing Time	0.973	0.4038	5 min	1.46	1.31	0.84	0.49	3.73
			12.5 min	0.65	0.35	0.58	0.30	1.14
			20 min	0.96	0.33	0.99	0.72	1.10
Fill Level	0.87	0.4421	25%	0.98	0.31	1.07	0.49	1.19
			40%	0.68	0.33	0.60	0.30	1.14
			55%	1.64	1.45	0.74	0.56	3.73
Rotational Speed	1.391	0.2836	10 rpm	1.54	1.34	0.79	0.58	3.73
			20 rpm	0.59	0.15	0.58	0.30	0.72
			30 rpm	0.95	0.26	0.96	0.56	1.19

* Statistically significant difference (*p* < 0.05)

## Data Availability

Data presented in this study is contained within the article. Further inquiries can be directed to the corresponding author.
